# ﻿Revision of the genus *Hemopsis* Kirti & Rose, 1987 (Lepidoptera, Crambidae), with descriptions of three new species from China

**DOI:** 10.3897/zookeys.1238.150678

**Published:** 2025-05-08

**Authors:** Cheng-Jun Yu, Xi-Cui Du

**Affiliations:** 1 College of Plant Protection, Southwest University, Chongqing, China Southwest University Chongqing China

**Keywords:** COI, DNA barcode, identification key, morphology, Spilomelinae, taxonomy

## Abstract

The genus *Hemopsis* is revised based on adult morphological characteristics and DNA barcodes. *Hemopsisabstracta***sp. nov.**, *H.coalita***sp. nov.**, and *H.heteroidea***sp. nov.** are described as new to science, and *Hemopsisdissipatalis* (Lederer, 1863) is DNA barcoded and redescribed based on new material from southern China. A key to *Hemopsis* species is given based on external morphology of adults and their genitalia characteristics. Images of adults and genitalia are provided.

## ﻿Introduction

The genus *Hemopsis* was erected by Kirti and Rose (1987) with *Botysdissipatalis* Lederer, 1863 as the type species, and *Botysangustalis* Snellen, 1890 was transferred to *Hemopsis* simultaneously. Both species were previously placed in the genus *Sy­llepte* Hübner, 1825 (= as misspelling *Sylepta*) by Hampson (1896). So far, *Hemopsis* is comprised of only these two species (Nuss et al. 2003–2024), and only *Hemopsisdissipatalis* has previously been recorded in China as “*Sylepta*” by Caradja (1925).

In appearance, the adults of *Hemopsis* species have faintly yellow wings with brown or fuscous markings, with an orbicular stigma, discoidal stigma, and postmedial line, which are common markings in Spilomelinae, as well as distinct broad brown bands along the outer margins of both wings. In addition, the reduced uncus in the male genitalia and the arc-shaped signum in the female genitalia are also markedly diagnostic characteristics of the genus.

Four *Hemopsis* species, including three new species from China, are recorded in the present study, with descriptions of their external and genitalia morphology.

## ﻿Materials and methods

### ﻿Taxon sampling

Specimens were collected by light trap at night and killed by ammonium hydroxide or ethyl acetate. Some specimens were soaked in anhydrous alcohol and stored at −20 °C or −80 °C in a refrigerator.

For phylogenetic analyses based on the mitochondrial COI barcode region, the outgroup taxon, *Nomophilanoctuella* (Denis & Schiffermüller, 1775), is very different from *Hemopsis* species in appearance. Based on the adult photos from the National Center for Biotechnology Information (NCBI) and description of the genital characteristics of *Ategumia* Amsel, 1956 (Kirti and Rose 1986: figs 4–7; Mally et al. 2019: fig. 13B), we found this genus was very similar to *Hemopsis* in external adult morphology and genitalia characteristics. Therefore, we chose the South American *Ategumiamatutinalis* (Guenée, 1854), the type species of *Ategumia*, as the ingroup taxon together with *Hemopsis* species.

The preparation of genitalia mainly follows Li and Zheng (1996). Morphological terminology mainly refers to Maes (1995) and Mally et al. (2019). The photographs of the adults were taken with a digital camera (Canon EOS 5D), and the genitalia photographs were obtained with a digital camera (Leica DFC 450) attached to a stereomicroscope (Leica M205 A).

The specimens, including the types of new species, are deposited in the Insect Collection, Southwest University, Chongqing, China (**SWU**). The type specimen photos of *Ategumiaadipaliskwantungialis* (Caradja) and *Ategumiaadipalisnigromarginalis* (Caradja), which we referred to in “Discussion”, are from the “Grigore Antipa” National Museum of Natural History, Bucharest, Romania (MNHGA).

### ﻿DNA extraction, PCR amplification, and sequencing

Three *Hemopsis* species and one outgroup species were sequenced in the present study. Total DNA was extracted from the dried legs using a TIANGEN DNA kit, following the manufacturer’s instructions. The mitochondrial COI gene’s 658-base-pair (bp) barcode region (Hajibabaei et al. 2006) was PCR amplified using the LepF1 and LepR1 primers. After verifying PCR products by running them on a 1% agarose gel, sequencing was conducted by Shanghai Sangon Biotech Co., Ltd (Shanghai, China) using the same primers as those used in PCR. In total, eight sequences from three *Hemopsis* species and three sequences from the outgroup species *Nomophilanoctuella* were obtained and uploaded to the NCBI. In addition, three sequences of another ingroup species, *Ategumiamatutinalis*, recorded as Ategumiasp.matutinalis DHJ03 in NCBI, were downloaded from the NCBI. Details of the specimens used for mitochondrial COI gene sequencing and phylogenetic analysis are provided in Table 1.

**Table 1.** Sample information for the ingroup and outgroup species included in the study.

Species	Sequence ID	Location	NCBI and BOLD accession no.
*Hemopsisdissipatalis*	YCJ23019	Yingzuijie, Hunan, China	PV186795
	YCJ23020	Huaping, Guangxi, China	PV186789
	YCJ23025	Qingshuitan, Guangxi, China	PV186790
	YCJ23026	Qingshuitan, Guangxi, China	PV186796
	YCJ23027	Qingshuitan, Guangxi, China	PV186791
*Hemopsisabstracta* sp. nov.	YCJ23036	Sudian Town, Yunnan, China	PV186793
	YCJ23037	Sudian Town, Yunnan, China	PV186794
*Hemopsiscoalita* sp. nov.	YCJ23033	Qingshuitan, Guangxi, China	PV186792
*Ategumiamatutinalis*	MHMYI426-10	Guanacaste, Costa Rica	JQ538928
	BLPCG605-08	Alajuela, Costa Rica	JQ563016
	BLPBA933-07	Guanacaste, Costa Rica	JQ572774
*Nomophilanoctuella*	IBILP890-19	Vimioso, Braganca, Portugal	OQ563609
	IBILP313-17	Tavira, Faro, Portugal	OQ563792
	YCJ23147	Youyang, Chongqing, China	PV196927

### ﻿Data analysis

The obtained sequences were searched for similarity using the BLAST tool of the NCBI database to compare nucleotide sequences between our specimens and the database. All COI sequences were manually aligned using MEGA X and then were translated into amino acid sequences for visual correction. Intraspecific and interspecific genetic distances were calculated using the Kimura 2-parameter (K2P) distance model (Kimura 1980). A phylogenetic tree was constructed using the maximum-likelihood (ML) method with 1,000 bootstrap replications (Stamatakis et al. 2008) based on the data of COI sequences. *Hemopsis* species and *Ategumiamatutinalis* were the ingroups, and *Nomophilanoctuella*, was regarded as the outgroup.

## ﻿Results

### ﻿DNA sequence analysis

**Figure 1.**
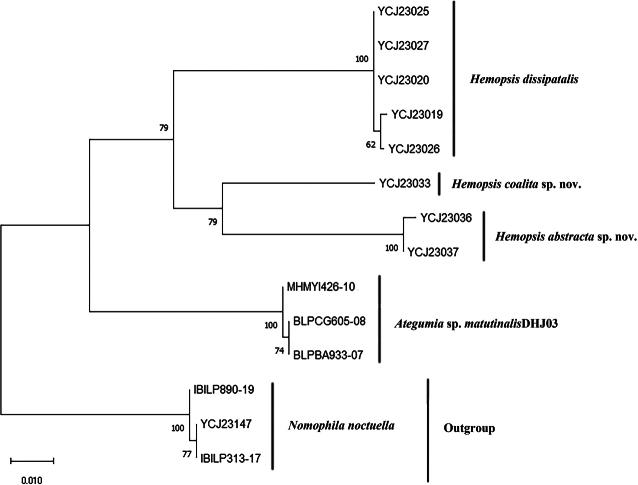
Phylogenetic hypothesis of relationships among ingroup and outgroup species inferred from a maximum-likelihood (ML) tree of the DNA barcode data. Numbers near the branches are bootstrap support values based on 1,000 replicates.

A total of 14 COI sequences, including three sequences of the outgroup species, were analysed. Three monophyletic clades for *Hemopsis* were observed in the phylogenetic tree and well separated from the outgroup species (Fig. 1). The pairwise genetic distances within and between these lineages are given in Table 2. In *Hemopsis*, the intraspecific genetic distance is 0.00%, and the interspecific genetic distance ranged from 6.84% to 8.15%. The genetic distances between the ingroup and outgroup species ranged from 9.12% to 10.41%. The maximum intraspecific COI genetic distance was much less than the minimum interspecific distance (Table 2). The results of the phylogenetic analysis were in full agreement with our morphological hypotheses for the investigated species.

**Table 2.** Kimura 2-parameter genetic distances (%) calculated within (in bold) and between the ingroup and outgroup species.

	1	2	3	4	5
1. *Hemopsisdissipatalis*	**0.00**				
2. *Hemopsiscoalita* sp. nov.	8.15	—			
3. *Hemopsisabstracta* sp. nov.	8.15	6.84	**0.00**		
4. *Ategumiamatutinalis*	8.64	8.81	10.03	**0.00**	
5. *Nomophilanoctuella*	10.21	9.57	10.41	9.12	**0.00**

### ﻿Taxonomic account

#### ﻿ Hemopsis

Taxon classificationAnimaliaLepidopteraCrambidae

Kirti & Rose, 1987

C0D710DB-AEA4-577F-9ECB-B70904C299FC

Hemopsis Kirti & Rose, 1987: 379. Type species: Botysdissipatalis Lederer, 1863, by original designation.

##### Diagnosis.

This genus is externally similar to *Mecyna* Doubleday, 1849 in appearance, but it can be distinguished by having the uncus reduced to a flat arch and by the long, balloon-shaped corpus bursae with a well-sclerotized arciform signum, while in *Mecyna*, the uncus is conical, and the corpus bursae is oval and has a longitudinal banded signum composed of granules. In addition, *Hemopsis* is very similar to *Ategumia* in external adult morphology and genitalia characteristics, but it can be distinguished by the forewing having the postmedial line excurved and merged with the outside broad band between wing veins M_2_ and CuA_2_; in *Ategumia*, the postmedial line of the forewing is slightly excurved and intersects with, or is separated from, the outside broad band between M_2_ and CuA_2_.

##### Redescription.

**Figure 2.**
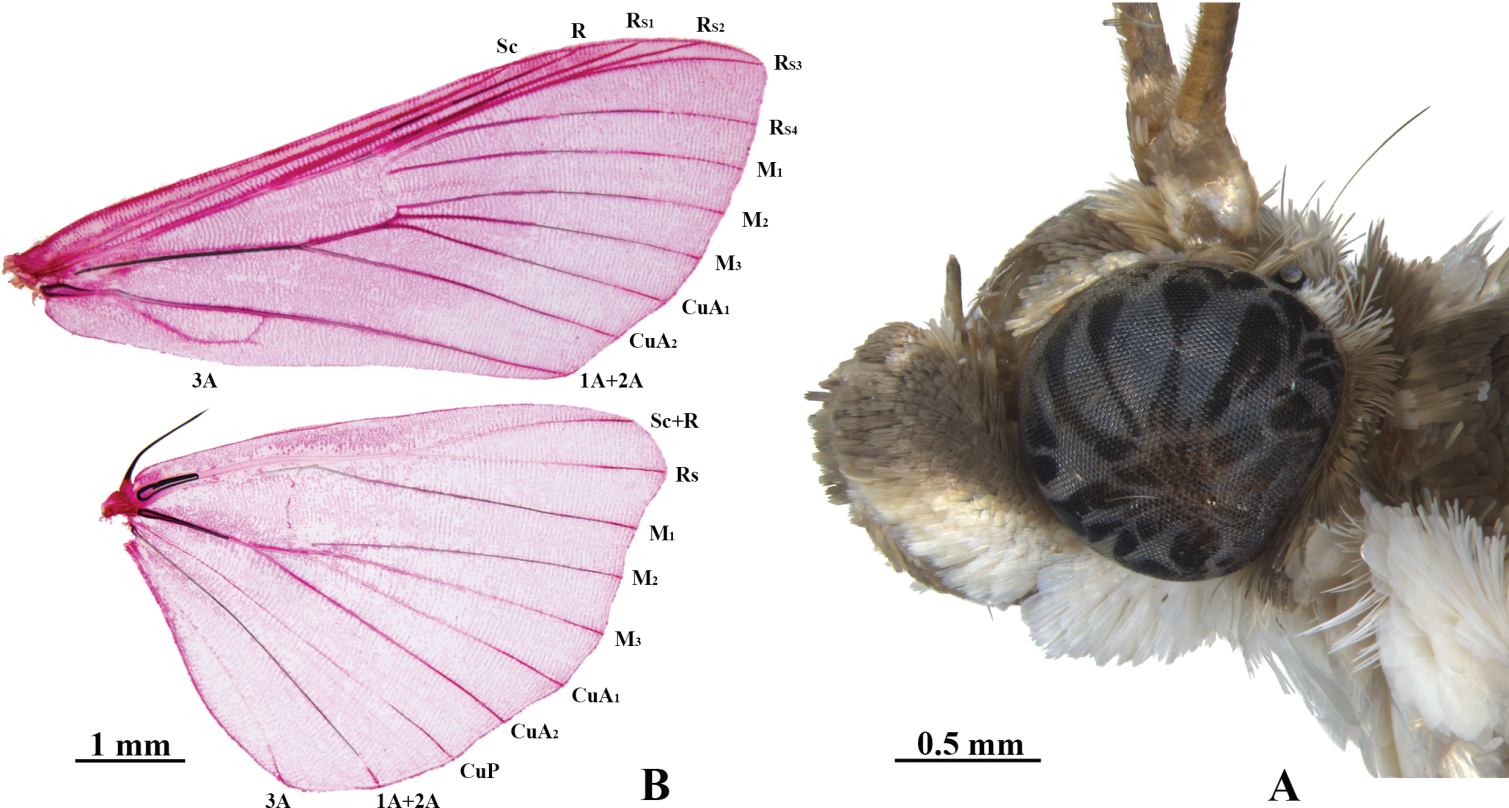
*Hemopsisdissipatalis*, male **A** head **B** wing venation, wing slide no. YCJ23003.

***Adult*.** Body yellowish brown; wings faintly yellow with brown or fuscous markings. Frons rounded. Antenna filiform; male with short cilia ventrally. Labial palpus obliquely upturned, with basal two-thirds white, brown distally; the third joint short, projecting forward (Fig. 2A). Maxillary palpus filiform. Forewing and hindwing with broad brown band along outer margin. Forewing with postmedial line from proximal two-thirds of costa and excurved between M_2_ and CuA_2_, then incurved to discoidal stigma below and sinuous to inner margin; cell somewhat less than half length of wing; R from cell at three-fourths above; R_S1_ very close to R_S2+S3_; R_S2_ anastomosed with R_S3_ about three-fifths beyond cell; R_S4_ slowly curved and close to R_S3+S4_ at base; M_2_, M_3_ and CuA_1_ from posterior angle of cell and uniformly spaced at the base; CuA_2_ from cell at three-quarters below. Hindwing with outer margin slightly protruded at Rs; cell less than one-third length of wing; discocellulars slightly incurved; Rs with one-quarter length anastomosed with Sc+R at base; M_2_, M_3_ and CuA_1_ from posterior angle of cell; CuA_1_ curved and approximated to M_3_ at base; CuA_2_ from cell at two-thirds below (Fig. 2B). Legs long and slender; middle tibia with inner spur about twice length of outer spur; hind tibia with outer proximal spurs one-third length of inner proximal spurs.

***Male genitalia*.** Uncus reduced to a flat arch; gnathos absent; valva tongue-shaped, bearing dense, long setae. Costa well sclerotized, inflated near base. Sacculus broad, protruded to base of fibula. Fibula hook-shaped, directed toward sacculus and distally curved toward costa. Saccus developed, triangular, with rounded end. Juxta U-shaped and sclerotized. Phallus cylindrical, with multiple cornuti.

***Female genitalia*.** Papillae anales densely setose. Antrum slightly sclerotized. Ductus bursae marked with well-developed and sclerotized colliculum. Ductus bursae shorter than corpus bursae. Corpus bursae long balloon-shaped, with a well-sclerotized, arciform signum.

##### Distribution.

China, India, Indonesia, Sri Lanka, Japan (Hampson 1896; Caradja 1925; Kirti and Rose 1987).

##### Remarks.

Although *Ategumia* is very similar to *Hemopsis* in external adult morphology and genitalia characteristics, we found they can be distinguished by the pattern of postmedial line of forewing. Moreover, they were well separated from each other in ML tree based on DNA barcodes.

### ﻿Key to *Hemopsis* species based on external and genitalia morphology

**Table:** 

1	Postmedial line of hindwing slightly excurved between M_1_ and CuA_2_ (Fig. 3A); phallus with a bunch of small spine-like cornuti, arranged in a curve (Fig. 4B)	***H.dissipatalis***
–	Postmedial line of hindwing distinctly excurved between M_2_ and CuA_2_ (Fig. 3B–D); phallus with two or three cornuti (Fig. 4D, F, H)	**2**
2	Postmedial line of hindwing well separated from outside broad band (Fig. 3B); corpus bursae with longitudinal arciform signum (Fig. 4J)	**3**
–	Postmedial line of hindwing closely accompanied by three small round spots which intersected with outside broad band (Fig. 3C, D); corpus bursae with transverse arciform signum (Fig. 4K, L)	**4**
3	Wings with postmedial lines somewhat punctated; male with black anal tuft	***H.angustalis***
–	Wings with postmedial lines smooth and somewhat thinner; male without black anal tuft (Fig. 3B)	***H.abstracta* sp. nov.**
4	Phallus with three connected cornuti, one short and lamellar, two long and banded (Fig. 4F)	***H.coalita* sp. nov.**
–	Phallus with two separate, banded cornuti, one long and wide, another thin and short (Fig. 4H)	***H.heteroidea* sp. nov.**

#### ﻿ Hemopsis dissipatalis

Taxon classificationAnimaliaLepidopteraCrambidae

(Lederer, 1863)

C5A6A2F2-379C-5E84-9CBE-4001A52982E4

Figs 3A , 4A, B, I

Botys dissipatalis Lederer, 1863: 474. Type locality: Indonesia (Ambon Island).
Sylepta dissipatalis : Hampson 1896: 335; Caradja 1925: 350.
Hemopsis dissipatalis : Kriti and Rose 1987: 380.

##### Material examined.

China – **Guangxi Zhuang Autonomous Region** • 1 ♂; Yangmeiao Scenic Spot, Huanjiang County; alt. 1160 m; 5 August 2022; Ci Tang & Shi-Qi Huang leg. • 3 ♂♂; Qingshuitan Protection Station, Rongshui County; alt. 374 m; 1, 3 August 2022; Ci Tang & Shi-Qi Huang leg. • 1 ♂; Protection Station, Huaping Natural Reservation Area; alt. 901 m; 3 August 2023; Shi-Qi Huang leg. • 3 ♂♂; Yinshan Park, Dayao Mountain; alt. 1100 m; 20 July 2015; Jing-Xia Zhao & Kai-Li Liu leg.; genitalia slide no.: YCJ23158, YCJ23225, YCJ23226 • 1 ♂; Hekou Protection Station, Dayao Mountain; alt. 1000 m; 18 July 2015; Jing-Xia Zhao & Kai-Li Liu leg.; genitalia slide no.: YCJ23221 • 1 ♂; Xiejiazhai, Huaping Nature Reserve, Guilin City; alt. 656 m; 14 June 2020; Hong Zhao leg.; genitalia slide no.: YCJ23157 – **Yunnan Prov.** • 2 ♀♀; Wangtianshu Scenic Spot, Xishuangbanna Dai Autonomous Prefecture; alt. 668 m; 31 July 2020;Yao Shen &Ci Tang leg.; genitalia slide no.: YCJ23197 • 2 ♀♀; Jinuo Township, Xishuangbanna Dai Autonomous Prefecture; alt. 1100 m; 15 May 2015; Xiao-Qiang Lu & Xi-Cui Du leg. • 2 ♂♂, 2 ♀♀; Caiyanghe River, Puer City; alt. 1500 m; 13 May 2015; Xiao-Qiang Lu & Xi-Cui Du leg. • 1 ♀; Taiyanghe River, Puer City; alt. 1659 m; 2 July 2021; Yao Shen & Ci Tang leg. – **Hunan Prov.** • 1 ♂, 2 ♀♀; Chenzhou Nature Reserve; alt. 1233 m; 3–4, June 2019; Xiao-Qiang Lu & Ying Yang leg.; genitalia slide no.: YCJ23196 ♀ • 1 ♂, 1 ♀; Dupangling Forest Farm; alt. 350 m; 1 August 2020; You Zeng leg.; genitalia slide no.: YCJ23109 ♀, YCJ23167 ♂ • 1 ♂; Guabangjiao, Yingzuijie National Nature Reserve; alt. 325 m; 13 July 2023; Shi-Qi Huang leg. – **Guangdong Prov.** • 1 ♂; Nanling Nature Reserve; alt. 1010 m; 26 July 2020; You Zeng leg.; genitalia slide no.: YCJ23108 – **Hainan Prov.** • 1 ♂; Wuzhishan Nature Reserve; alt. 745 m; 27 March 2021; Yao Shen leg.; genitalia slide no.: YCJ23166.

##### Redescription.

**Figure 3.**
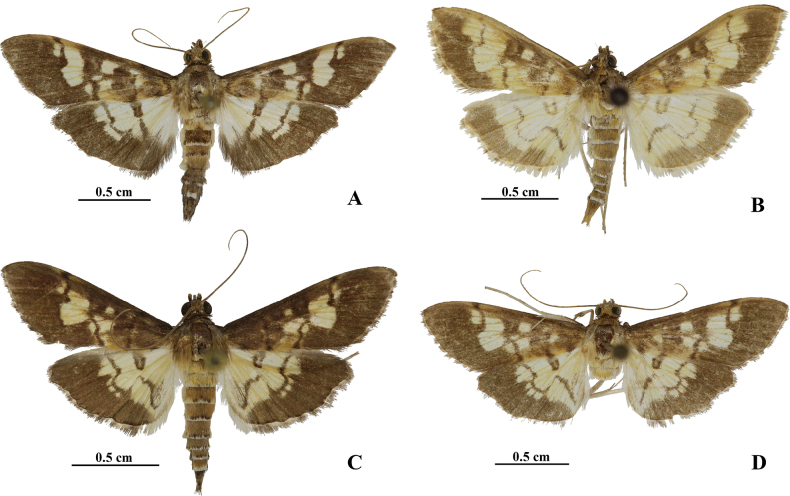
Adults of *Hemopsis* species, male **A***H.dissipatalis***B***H.abstracta* sp. nov., holotype **C***H.coalita* sp. nov., holotype **D***H.heteroidea* sp. nov., holotype.

***Adult*** (Fig. 3A). Wingspan 22.0–32.0 mm, forewing length 10.0–16.0 mm. Frons brown. Vertex brown anteriorly, yellowish white posteriorly. Antenna yellowish brown; ventral cilia about one-third length of flagellomeres diameter in male. Maxillary palpus black-brown, white at base. Patagium brown. Tegula light brown, yellowish white distally. Forewing and hindwing pale yellow, with fuscous stigmas, lines, and broad bands along outer margin. Forewing diffused fuscous at base, proximal two-thirds of costal and broad band along outer margin fuscous; orbicular stigma a patch; discoidal stigma quadrilateral, extended to costal; antemedial line closely accompanied by a fuscous square spot below orbicular stigma; postmedial line distinctly excurved and merged with outside broad band between M_2_ and CuA_2_. Hindwing with discoidal stigma reniform; postmedial line slightly excurved between M_2_ and CuA_2_, then straight to discoidal stigma below and nearly straight downward, connected to outside broad band at CuA_2_ and merged into outside broad band after CuP. Cilia brown, mixed with some white. Legs off-white, front coax and femur light brown; middle tibia brown; hind tibia with outer distal spurs half length of inner distal spurs. Abdomen dorsally yellowish brown and terminally diffused white and black at each segment, white ventrally.

**Figure 4.**
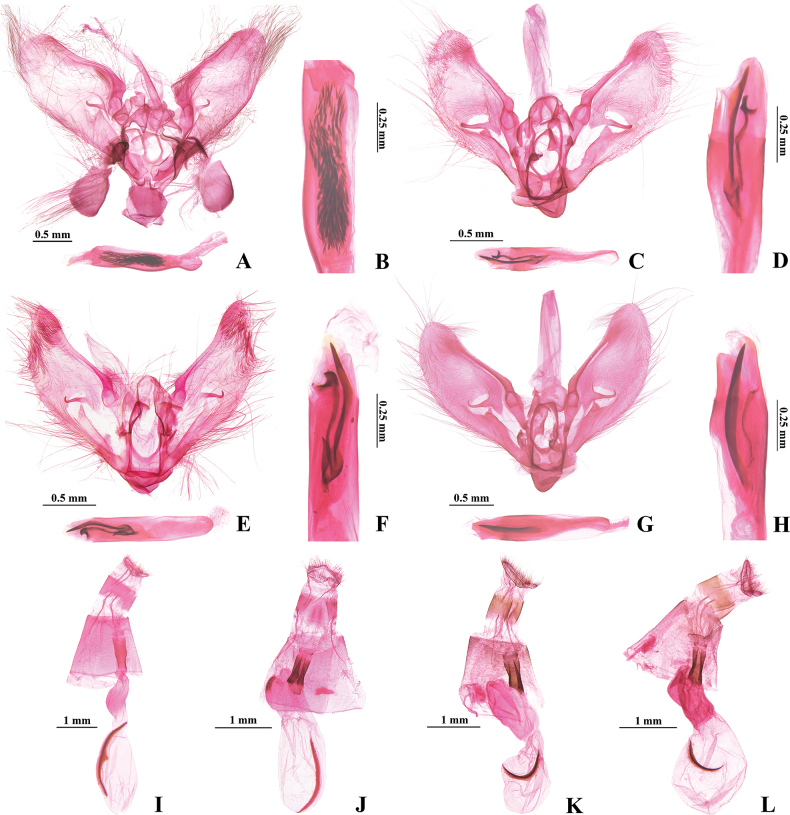
Genitalia of *Hemopsis* species **A, B, I***H.dissipatalis***C, D, J***H.abstracta* sp. nov. **E, F, K***H.coalita* sp. nov. **G, H, L***H.heteroidea* sp. nov. **A, C, E, G** male **B, D, F, H** cornuti **I, J, K, L** female. Slide no.: **A, B** YCJ23225 **C, D** YCJ23137 **E, F** YCJ23231 **G** YCJ23162 **H** YCJ23233 **I** YCJ23196 **J** YCJ23107 **K** YCJ23223 **L** YCJ23224.

***Male genitalia*** (Fig. 4A, B). Valva very gradually narrowed distally, distal end rounded; fibula short and small. Phallus with a bunch of small, spine-like cornuti arranged in a curve.

***Female genitalia*** (Fig. 4I). Apophyses anteriores about two times length of apophyses posteriores. Ductus bursae approximately one-quarter length of corpus bursae. Corpus bursae elliptical anteriorly, posterior third distinctly narrowed and weakly sclerotized; arciform signum longitudinal, with a hill-like protrusion medially.

##### Distribution.

China (Chongqing, Hunan, Yunnan, Guangxi, Guangdong, Hainan, Fujian, Taiwan), Indonesia, India, Sri Lanka, Japan (Hampson 1896; Caradja 1925; Kirti and Rose 1987).

##### Remarks.

Hemopsisnr.dissipatalis of Matsui et al. (2022) is quite different from *H.dissipatalis* (Lederer, 1863) described and illustrated by Kirti and Rose (1987). According to Matsui et al. (2022), *H.dissipatalis* identified in Japan, is characterized by the conical uncus and the corpus bursae having a transverse signum. However, according to the description and illustration by Kirti and Rose (1987), the uncus of *Hemopsis* species is reduced and the female genitalia of *H.dissipatalis* has a longitudinal signum. In addition, we found H.nr.dissipatalis illustrated by Matsui et al. (2022; supplementary file S1) has a spindle-shaped valva; the fibula is well developed, with its tip curved downward toward sacculus; and the phallus has claw-like, larger, leaf-like cornuti. However, in *H.dissipatalis*, the valva is tongue-shaped; the fibula is short and small, tip curved upward toward the costa; and the phallus has a bunch of small, spine-like cornuti. Therefore, we do not consider H.nr.dissipatalis of Matsui et al. (2022) to be a species of *Hemopsis*.

#### ﻿ Hemopsis abstracta sp. nov.

Taxon classificationAnimaliaLepidopteraCrambidae

1FCACDE9-0DFB-52E4-81C0-A6BC4EC27225

https://zoobank.org/D970327C-1B87-4060-874C-84751DF70932

Figs 3B , 4C, D, J

##### Type material.

***Holotype***: China • ♂; pinned, with genitalia on a separate slide; **Yunnan Prov.**, Dawei Mountain, Honghe Prefecture; alt. 2700 m; 27 May 2018; Xiao-Qiang Lu & Xi-Cui Du leg.; genitalia slide no.: YCJ23137. ***Paratypes***: pinned, some with genitalia on separate slides, respectively. China – **Yunnan Prov.** • 5 ♂♂, 2 ♀♀; other data same as the holotype. • 11 ♂♂, 2 ♀♀; Dawei Mountain, Honghe Prefecture; alt. 2363 m; 17 July 2015; Xue-Li Wei leg.; genitalia slide no.: YCJ23106 ♀, YCJ23107 ♀, YCJ23125 ♀ • 4 ♂♂; Huanglian Mountain, Honghe Prefecture; alt. 900 m; 23 May 2018; Xiao-Qiang Lu & Xi-Cui Du leg. • 1 ♂, 1 ♀; Jingdong County; alt. 22 August 2009; Xi-Cui Du leg. • 4 ♂♂; Sudian Town, Dehong prefecture; alt. 1947 m; 15 June 2020; Ying Yang & Hong Zhao leg.; genitalia slide no.: YCJ23158 • 1 ♂; Daxichang, Malipo County; alt. 1465 m; 5 June 2015; Man-Fei Tao leg. • 1 ♀; Baihua Ridge, Baoshan City; alt. 1520 m; 13 August 2007; Dan-Dan Zhang leg. • 2 ♂♂; Cuanlong Village, Mangbang Town, Tengchong County; alt. 1329 m; 9 August 2015; Jing-Xia Zhao & Hao Wei leg. • 2 ♂♂; Caiyanghe River, Puer City; alt. 1500 m; 13 May 2015; Xiao-Qiang Lu & Xi-Cui Du leg. – **Guangxi Zhuang Autonomous Region** • 6 ♂♂, 3 ♀♀; Cenwanglaoshan, Dalongping Protection Station; alt. 1290 m; 5 August 2014; Xue-Li Wei & Chao Ran leg.; genitalia slide no.: YCJ23110 ♂, YCJ23220 ♂, YCJ23222 ♂.

##### Diagnosis.

This species is similar to *H.angustalis* in appearance, but it can be distinguished by its wings which have relatively larger stigmas, and the centrally lighter brown discoidal stigma; postmedial lines are smooth and somewhat thinner; the male lack a black anal tuft; the phallus has three anteriorly connected cornuti, one short and laminar and two long bands (one curved and distally bilobed and the other relatively straight, longer and distally pointed). In *H.angustalis*, stigmas are relatively smaller, and the discoidal stigma is uniformly fuscous; postmedial lines are somewhat punctated and thicker; the male has a black anal tuft; the phallus has three cornuti, with one small and spine-like and two long bands (one curved and the other relatively straight, longer and distally pointed) (Kirti and Rose 1987: fig. 6).

##### Description.

***Adult*** (Fig. 3B). Wingspan 23.0–27.0 mm, forewing length 12.0–14.0 mm. Frons brown. Vertex yellowish brown. Antenna yellowish brown, ventral cilia about one-quarter length of flagellomeres diameter in male. Maxillary palpus brown, white at base. Patagium yellowish brown. Tegula light brown, distally yellowish white. Forewing and hindwing pale yellow, with stigmas, lines, and broad bands along outer margin brown. Forewing diffused brown at base and proximal half of costal; orbicular stigma nearly round, sometimes light centrally; discoidal stigma reniform, centrally light; antemedial line closely accompanied by a brown square spot below orbicular stigma; postmedial line distinctly excurved and merged with outside broad band between M_2_ and CuA_2_. Hindwing with discoidal stigma reniform, centrally pale yellow; postmedial line distinctly excurved between M_2_ and CuA_2_, then incurved to discoidal stigma below and curved down to posterior margin, well separated from outer broad band; outer broad band narrowed at middle. Cilia pale yellow, with brown basal line. Legs off-white; front coax and femur light brown; tibia distally brown; hind tibia with outer distal spurs two-thirds length of inner distal spurs. Abdomen dorsally yellowish brown and terminally white at each segment; ventrally white.

***Male genitalia*** (Fig. 4C, D). Valva very gradually narrowed distally, distal end rounded. Fibula comparatively well developed, with tip sharp. Phallus with three anteriorly connected cornuti: one short lamina and two long bands (one curved and distally bilobed and the other relatively straight, longer, and distally pointed).

***Female genitalia*** (Fig. 4J). Apophyses anteriores about 1½ times length of apophyses posteriores. Ductus bursae approximately one-third length of corpus bursae. Corpus bursae elliptical anteriorly, posterior third slightly narrowed and weakly sclerotized; arciform signum longitudinal.

##### Etymology.

The specific name is derived from the Latin *abstractus*, meaning separated, in reference to the postmedial line of the hindwing well separated from the outer broad band.

##### Distribution.

China (Yunnan, Guangxi).

#### ﻿ Hemopsis coalita sp. nov.

Taxon classificationAnimaliaLepidopteraCrambidae

3FE50D02-AF76-5333-9724-FC51B3346914

https://zoobank.org/CA8C607E-0C45-4094-8340-04AE5A087C86

Figs 3C , 4E, F, K

##### Type material.

***Holotype***: China • ♂; pinned, with genitalia on a separate slide; **Guangxi Zhuang Autonomous Region**, Qingshuitan Protection Station, Rongshui County; alt. 374 m; 1 August 2022; Ci Tang & Shi-Qi Huang leg.; genitalia slide no.: YCJ23229. ***Paratypes***: pinned, some with genitalia on separate slides, respectively. China – **Guangxi Zhuang Autonomous Region** • 1 ♀; Yangmeiao Scenic Spot, Huanjiang County; alt. 1160 m; 5 August 2022; Ci Tang & Shi-Qi Huang leg. genitalia slide no.: YCJ23223 • 1 ♀; Xiaosang Village, Rongshui County; alt. 2000 m; 30 July 2015; Ji-Ping Wan leg.; genitalia slide no.: YCJ23237 – **Hainan Prov.** • 1 ♂; Bawangling National Nature Reserve; 14 June 2010; Li Kang leg.; genitalia slide no.: YCJ23231 • 1 ♂; Wuzhi Mountain; alt. 745 m; 27 March 2021; Li Kang leg.; genitalia slide no.: YCJ23236.

##### Diagnosis.

This species is very similar to *H.dissipatalis* in appearance, but it can be distinguished in having the hindwing with the postmedial line distinctly excurved between M_2_ and CuA_2_ and closely accompanied by three pale yellow spots outside; the valva with its distal end nearly triangular and apically pointed; the phallus with three anteriorly connected cornuti; the corpus bursae with its posterior half cystiform and slightly narrower than the anterior half; and a transverse arciform signum. While in *H.dissipatalis*, the postmedial line of hindwing is slightly excurved between M_2_ and CuA_2_ and has no spot accompanied outside; the valva is very gradually distally narrowed and apically rounded; the phallus has a bunch of small, spine-like cornuti; the posterior third of corpus bursae is distinctly narrowed; and the arciform signum is longitudinal. In addition, male genitalia of *H.coalita* are similar to *H.abstracta*, especially in the shape of the cornutus, but *H.coalita* can be differentiated by the valva which has its distal end nearly triangular and apically pointed, the corpus bursae which is distinctly medially constricted, and the transverse arciform signum. In *H.abstracta*, the distal end of valva is nearly semicircular and apical end rounded, the corpus bursae is slightly posteriorly constricted, and arciform signum is longitudinal.

##### Description.

***Adult*** (Fig. 3C). Wingspan 22.0–28.0 mm, forewing length 12.0–14.0 mm. Frons brown. Vertex yellowish brown. Antenna yellowish brown; ventral cilia about one-quarter length of flagellomeres diameter in male. Maxillary palpus brown, white at base. Patagium yellowish brown. Tegula light brown, distally yellowish white. Forewing and hindwing pale yellow, with stigmas, lines, and broad bands along outer margin fuscous. Forewing diffused fuscous at base one-third; costal fuscous, pale near postmedial line; orbicular stigma nearly round; discoidal stigma reniform; antemedial line closely accompanied by a square spot below orbicular stigma; postmedial line distinctly excurved and merged with outside broad band between M_2_ and CuA_2_. Hindwing with discoidal stigma reniform, centrally pale yellow; postmedial line distinctly excurved between M_2_ and CuA_2_, closely accompanied by three pale yellow spots intersected with outside broad band, then straight to discoidal stigma below and curved down to posterior margin. Cilia fuscous, with pale-yellow basal line. Legs off-white, front coax and femur light brown, tibia distally brown; hind tibia with outer distal spurs two-thirds length of inner distal spurs. Abdomen yellowish-brown dorsally and terminally white mixed black at each segment, ventrally white.

***Male genitalia*** (Fig. 4E, F). Valva gradually narrowed distally, with distal end nearly triangular and apical end pointed; fibula slender. Phallus with three anteriorly connected cornuti, one short lamina, and two long bands (one curved, distally bilobed and the other relatively straight, longer, and distally pointed band).

***Female genitalia*** (Fig. 4K). Apophyses anteriores about two times length of apophyses posteriores. Ductus bursae approximately one-third length of corpus bursae. Corpus bursae with anterior half nearly elliptical, posterior half cystiform and weakly sclerotized, distinctly constricted medially; arciform signum transverse.

##### Etymology.

The specific name is derived from the Latin *coalitus*, meaning connective, in reference to the three connected cornuti.

##### Distribution.

China (Guangxi, Hainan).

#### ﻿ Hemopsis heteroidea sp. nov.

Taxon classificationAnimaliaLepidopteraCrambidae

2D152BA5-3746-58BC-9C50-4F45AB6827A3

https://zoobank.org/DF71A32B-7E4C-4E5C-A7E9-33E3D5332431

Figs 3D , 4G, H, L

##### Type material.

***Holotype***: China • ♂; pinned, with genitalia on a separate slide; **Chongqing Municipality**, Tudiyan, Simian Mountain; alt. 1200 m; 9 August 2011; Gui-Qing Gui & Li-Fang Song leg.; genitalia slide no.: YCJ23162. ***Paratypes***: pinned, some with genitalia on separate slides, respectively. CHINA – **Chongqing Municipality** • 10 ♂♂; other data same as the holotype, genitalia slide no.: YCJ23155, YCJ23161 • 3 ♂♂; same locality; 15 July 2012; Gui-Qing He et al. leg. • 12 ♂♂; Tudiyan, Simian Mountain; alt. 1156 m; 27 August 2019; Hong Zhao & Xi-Cui Du leg. genitalia slide no.: YCJ23159 • 3 ♂♂; Simian Mountain; alt. 1000 m; 22 July 2010; Li-Fang Song & Xi-Cui Du leg.; genitalia slide no.: YCJ23156, YCJ23160 • 1 ♂, 1 ♀; Simian Mountain; alt. 902 m; 26 July 2018; Xiao-Qiang Lu & You Zeng leg. • 3 ♂♂; Dahonghai, Simian Mountain; alt. 1250 m; 14 August 2011; Gui-Qing He & Li-Fang Song leg. • 1 ♂, 3 ♀♀; Dawopu, Simian Mountain; alt. 902 m; 10 September 2016; Xue-Li Wei & Qiu-Long Yang leg.; genitalia slide no.: YCJ23158 ♂ • 1 ♀; Dawopu, Simian Mountain; alt. 1003 m; 21 July 2017; Yi Long & Qiu-Long Yang leg.; genitalia slide no.: YCJ23105 • 1 ♂; Dawopu, Simian Mountain; alt. 1183 m; 29 June 2019; Yao Shen & Xi-Cui Du leg. • 3 ♀♀; Jinfo Mountain; alt. 891 m; 14 July 2017; Yi Long & Mao-Jun Yuan leg. – **Guangxi Zhuang Autonomous Region** • 2 ♂♂; Yuxi Protection Station, Luocheng County; alt. 353 m; 12 August 2022; Ci Tang & Shi-Qi Huang leg.; genitalia slide no.: YCJ23234 • 2 ♀♀; Pingying Village, Luocheng County; alt. 470 m; 10 August 2022; Ci Tang & Shi-Qi Huang leg. • 1 ♀; Yangmeiao, Huanjiang County; alt. 1160 m; 5 August 2022; Ci Tang & Shi-Qi Huang leg. ; genitalia slide no.: YCJ23223 • 1 ♀; Gaozhai Village, Maoer Mountain, Guilin City; alt. 1100 m; 24 July 2015; Kai-Li Liu & Jing-Xia Zhao leg.; genitalia slide no.: YCJ23224 – **Guangdong Prov.** • 1 ♂; Nanling Nature Reserve; alt. 1010 m; 27 July 2020; You Zeng leg.; genitalia slide no.: YCJ23230 • 1 ♂; Babaoshan Protection Station, Nanling Nature Reserve; alt. 1070 m; 23 August 2010; Xi-Cui Du leg. genitalia slide no.: YCJ23232 • 1 ♂; Dadong Mountain, Lianzhou City; alt. 650 m; 25 June 2014; Dan-Dan Zhang leg. – **Hunan Prov.** • 1 ♂; Babaoshan Protection Station, Nanling Nature Reserve; alt. 1021 m; 9 September 2020; Hong Zhao leg.; genitalia slide no.: YCJ23235.

##### Diagnosis.

Externally this species is very similar to *H.coalita* sp. nov. and difficult to distinguish from each other except by genital characteristics. In *H.heteroidea*, the distal end of valva is nearly semicircular and the apical end is rounded; the phallus has two separate, banded cornuti; one is long and wide and the other is thin and short. The posterior half of corpus bursae is narrowed and about half the width of the anterior half. In *H.coalita*, the distal end of the valva is nearly triangular and the apical end is pointed; the phallus has three anteriorly connected cornuti, one short lamina, and two long bands; and the posterior half of the corpus bursae is cystiform and slightly narrower than the width of anterior half.

##### Description.

***Adult*** (Fig. 3D). Refer to the adult description of *H.coalita* because it is externally identical.

***Male genitalia*** (Fig. 4G, H). Valva very gradually narrowed distally, distal end nearly semicircular and apical end rounded; fibula slender. Phallus with two separate, banded cornuti, one long, wide, and distally pointed, the other thin and short.

***Female genitalia*** (Fig. 4L). Apophyses anteriores about two times length of apophyses posteriores. Ductus bursae approximately one-quarter length of corpus bursae. Corpus bursae with anterior half nearly oval, posterior half sclerotized and narrowed, about half width of the anterior half; arciform signum transverse.

##### Etymology.

The specific name is derived from the Latin *heteroideus*, meaning different shapes, in reference to two different banded cornuti.

##### Distribution.

China (Chongqing, Hunan, Guangxi, Guangdong).

## ﻿Discussion

The reduced uncus in the male genitalia in *Hemopsis* is also found in *Ategumia*, *Bocchoris* Moore, 1885, *Diasemia* Hübner, 1825, and *Choristostigma* Warren, 1892. Among these genera, *Ategumia*, *Bocchoris*, and *Diasemia* belong to Nomophilini (Mally et al. 2019). We found that the wing markings of *Hemopsis* were very similar to that of *Ategumia* and *Mecyna* in the tribe Nomophilini. Meanwhile, in *Hemopsis*, the reduced uncus, tongue-shaped valva, fibula directed toward sacculus, multiple cornuti, strongly sclerotized colliculum, slightly sclerotized antrum, and a transverse or longitudinal elongate signum are all consistent with the characteristics of the tribe Nomophilini. Therefore, we place the genus *Hemopsis* in the tribe Nomophilini.

Two subspecies of *Ategumiaadipalis* (Lederer, 1863), *A.a.kwantungialis* (Caradja, 1925) and *A.a.nigromarginalis* (Caradja, 1925), are very similar to *Hemopsis* species in appearance. Based on the original descriptions and type specimen photos of these two subspecies, we found *A.a.kwantungialis* was nearly identical with Hemopsisnr.dissipatalis of Matsui et al. (2022) in appearance. Moreover, according to our examination of *Ategumiaadipalis* and description by Kirti and Rose (1986), we do not think that *A.a.kwantungialis* is a subspecies of *A.adipalis*. Furthermore, we found that the lectotype specimen (MNHGA 177.705) of *A.a.nigromarginalis* was externally similar to *H.dissipatalis* and had some inconspicuous difference in wing markings, the allolectotype specimen (MNHGA 177.706) was externally similar to *H.abstracta* sp. nov. and had some inconspicuous difference in wing markings, and the paralectotype specimen (MNHGA 177.707) was more similar to *H.coalita* sp. nov. and *H.heteroidea* sp. nov. in appearance than to *A.adipalis*. Considering these observations and the original descriptions, we think that *A.a.nigromarginalis* is incorrectly associated as a subspecies of *A.adipalis* and that it may be misplaced in the genus *Ategumia* and may belong to *Hemopsis* instead. Therefore, further studies of these species and subspecies are needed to elucidate their generic placement and relationships among them.

## Supplementary Material

XML Treatment for Hemopsis

XML Treatment for Hemopsis dissipatalis

XML Treatment for Hemopsis abstracta

XML Treatment for Hemopsis coalita

XML Treatment for Hemopsis heteroidea

## ﻿Additional information

### Author ORCIDs

Xi-Cui Du https://orcid.org/0000-0002-7796-7303

### Data availability

All of the data that support the findings of this study are available in the main text.

## References

[B1] Caradja AV (1925) Ueber Chinas Pyraliden, Tortriciden, Tineiden nebst kurze Betrachtungen, zu denen das Studium dieser Fauna Veranlassung gibt (Eine biogeographische Skizze). Memoriile Sectiunii Stiintifice. Academia Romana (ser. 3), Bucuresti 3(7): 257–383. [pls 1–2]

[B2] Hajibabaei M Janzen DH Burns JM Hallwachs W Hebert PDN (2006) DNA barcodes distinguish species of tropical Lepidoptera. Proceedings of the National Academy of Sciences of the United States of America 4(103): 968–971. 10.1073/pnas.0510466103 PMC1327734 16418261

[B3] Hampson GF (1896) The Fauna of British India, Including Ceylon and Burma. Moths. Vol. IV. Taylor and Francis, London, Thacker, Spink & Co., Calcutta, Thacker & Co., Bombay and R. Friedländer & Sohn, Berlin, xxviii + 594 pp. 10.5962/bhl.title.100745

[B4] Kimura M (1980) A simple method for estimating evolutionary rates of base substitutions through comparative studies of nucleotide sequences. Journal of Molecular Evolution 16: 111–120. 10.1007/BF01731581 7463489

[B5] Kirti JS Rose HS (1986) Comments on the genus *Bocchoris* Moore along with the description of new a genus *Nanakogobinda* (Pyraustinae: Pyralidae: Lepidoptera). Journal of Entomological Research 10(1): 63–68.

[B6] Kirti JS Rose HS (1987) Taxonomic status of two north-eastern India species referred to genus *Sylepta* Hubner with the proposal of a new genus *Hemopsis*. Entomon 12(4): 379–383.

[B7] Lederer J (1863) Beitrag zur Kenntniss der Pyralidinen. Wiener Entomologische Monatschrift 7 (8, 10–12): 243–280, 331–504. [pls 2–18]

[B8] Li HH Zheng ZM (1996) Methods and techniques of specimens of Microlepidoptera. Journal of Shaanxi Normal University (natural science edition) 24(3): 63–70.

[B9] Maes K (1995) A comparative morphological study of the adult Crambidae (Lepidoptera: Pyraloidea). Bulletin et Annales de la Société Royale Belge d’Entomologie 131: 383–434.

[B10] Mally R Hayden JE Neinhuis C Jordal BH Nuss M (2019) The phylogenetic systematics of Spilomelinae and Pyraustinae (Lepidoptera: Pyraloidea: Crambidae) inferred from DNA and morphology. Arthropod Systematics & Phylogeny 77(1): 141–204. 10.26049/ASP77-1-2019-07

[B11] Matsui Y Mally R Kohama S Aoki I Azuma M Naka H (2022) Molecular phylogenetics and tribal classification of Japanese Pyraustinae and Spilomelinae (Lepidoptera: Crambidae). Insect Systematics & Evolution 54: 77–106. 10.1163/1876312X-bja10037

[B12] Nuss M Landry B Mally R Vegliante F Tränkner A Bauer F Hayden J Segerer A Schouten R Li H Trofimova T Solis MA Prins De J Speidel W (2003–2024) Global Information System on Pyraloidea. http://www.pyraloidea.org [Accessed on: 2025-4-15]

[B13] Snellen PCT (1890) A catalogue of the Pyralidina of Sikkim collected by Henry J. Elwes and the late Otto Möller, with notes by H. J. Elwes. Transactions of the Entomological Society of London, 557–647. [pls 19–20] 10.1111/j.1365-2311.1890.tb03031.x

[B14] Stamatakis A Hoover P Rougemont J (2008) A rapid bootstrap algorithm for the RAxML Web Servers. Systematic Biology 57: 758–771. 10.1080/10635150802429642 18853362

